# Report of the Pennsylvania Hospital for the Insane

**Published:** 1855-03

**Authors:** 


*Report of the Pennsylvania Hospital for the Insane.* By Thomas S. Kirkbridge, M. D., Physician to the Institution. Philadelphia: 1855

This Institution, under the direction of Dr. Kirkbridge, whose labors for the relief of the unfortunate subjects of insanity have brought him so favorably before the country, contained an average of 229 patients during the past year; 198 were admitted, and 190 discharged or died, of whom 98 were *cured;* 32 much *improved;* 19 *improved;* 15 *stationary;* 26 *died.*

